# Hospice de Chinon

**Published:** 1832-08

**Authors:** 


### HOSPICE DE CHINON.

*Emphysema occurring in a Consumptive Patient, after the Application of Leeches on the Parietes of the Chest.* Communicated by MM. Desmée and Gendron.*

Martin Forgette, a husbandman, twenty years of age, and of a delicate constitution, was admitted into the Hospice de Chinon on the 12th of December, 1831. He suffered from an affection of the chest; had had cough for more than six months, the sputa purulent, and often mingled with blood. A violent fever reduced him greatly; and percussion and auscultation left no doubt that tuberculous phthisis was present, and that the left lung was more especially diseased. The man, previous to his admission, had oftentimes suffered much from intense pains in the chest, which now returning, and the right side being most acutely affected, six leeches were, on the 9th of January, ordered to be applied, and the application was followed with decided relief. But the same evening, a notable swelling of the thoracic parietes was perceived, which, beginning on the side where the leeches had been applied, rapidly spread to the other, and quickly extended to the neck and face, and became so serious as greatly to alarm the patient, and threaten him with immediate suffocation.

The diagnosis was easy: the elastic tension of the integuments of the chest, distended both on the sides and in front, sonorous on percussion, and crepitating under pressure, proved that the sudden tumefaction was due to an escape of air, or a developement of gas, in the subcutaneous cellular tissue.

Cupping glasses were applied over the leech-bites, but without any good effect; frictions were also instituted.

January 11, MM. Lafon and Gendron met in consultation, and decided on evacuating the effused fluid by several punctures. The gases escaped with a slight hissing noise, and forming little bubbles: and the discharge was rendered more free by directing the pressure towards the punctures which had been made. Notwithstanding what was done, the patient felt very little relief, and the emphysema extended to the abdominal parietes, and also to the arms and legs.

January 13. Scarifications were again resorted to, and the punctures being deeper, the swelling was very considerably re-

* Gazette Medicale.

duced, and completely disappeared in the course of four days: but the patient's state became more and more hopeless each succeeding day, and his respiration more and more laborious. On the 26th of January, he was himself conscious of his approaching dissolution, saying to the attendants that day would be his last, and towards the evening he died.

The autopsy shewed considerable derangement of the thoracic viscera. Numerous adhesions were found uniting the pulmonary and costal pleuras, especially on the left side: false membranes hung from many parts, and appeared to be filled with bubbles of air. The left lung was hepatised and studded with tubercles, and contained several vomicæ, of which the largest was towards the apex. The right lung still crepitated throughout a considerable part of its extent, but it likewise was tuberculated, and contained several cavities, though of less size. The pericardium was filled with a yellowish serum. The heart larger than in a healthy state.

To what cause can we attribute the emphysema here observed? Should it be regarded as of spontaneous origin, or as solely due to the leechbites? May we not believe that a rupture had occurred in a violent paroxysm of coughing, (and the subtraction of blood, in lessening the strength of the cohesions, might have favored it,) the pleura pulmonalis and the pleura costalis being united, and forming, as it were, one of the walls of a tuberculous excavation; and that, by this opening, the air entering the lung became extravasated into the exterior cellular tissue; and thus, that the emphysema was produced? As to the false membranes, so dense and highly organized, may we not consider them as an admirable effort of nature to repair the deranged structure; and, in a state of organization more perfect, might they not act in concurrence and as a kind of appendages to the lungs?

---

